# Analysis on the temporal and spatial characteristics of the shallow soil temperature of the Qinghai-Tibet Plateau

**DOI:** 10.1038/s41598-022-23548-4

**Published:** 2022-11-17

**Authors:** Yujie Li, Cunjie Zhang, Zhenchao Li, Liwei Yang, Xiao Jin, Xiaoqing Gao

**Affiliations:** 1 Key Laboratory of Land Surface Process and Climate Change in Cold and Arid Regions of Chinese Academy of Sciences, Northwest Institute of Eco-Environment and Resources, Chinese Academy of Sciences, 320, Donggang West Road, Lanzhou, 730000 Gansu China; 2 University of Chinese Academy of Sciences, Beijing, 100049 China; 3 China Meteorological Administration, National Climate Center, Beijing, China

**Keywords:** Climate-change impacts, Atmospheric dynamics

## Abstract

Shallow soil refers to the soil layer within the 50 cm depth. Shallow soil temperature (ST) directly or indirectly affects many processes in the soil, such as seed germination, plant growth, and water evaporation. Therefore, the study of shallow ST is of great significance in understanding the surface energy, water cycle, ecology and climate change. This work collected observational data from 141 meteorological stations on the Qinghai-Tibet Plateau from 1981 to 2020 and ERA5 reanalysis data, used the “Moving Surface Spline Interpolation Algorithm Based on Green’s Function” and “Fuzzy C-means algorithm”, and analyzed the temporal and spatial change characteristics of ST at different levels. The results showed that 1) the temperature increase of 0–20 cm (the surface layer of the shallow soil) was roughly the same. The average annual ST was 9.15–9.57°, and the interdecadal variabilities were 0.49–0.53 K/10a. The average annual ST of 40 cm (the bottom layer) was 8.69°, and the interdecadal variability reached 0.98 K/10a. 2) Considering the 7 regions, the warming trend was obvious, and there were certain regional differences. The average annual ST in different regions ranged from 5.2 (northeastern Plateau) to 17.1 °C (western Sichuan Plateau), with a difference of nearly 12 K. The standard deviation ranged from 0.40 (western Sichuan Plateau) to 0.61 K (Qiangtang Plateau), with a difference of 0.21 K. 3) The errors of the obtained grid data were basically less than 3%, which were much smaller than the errors obtained from the ERA5 reanalysis data. This work is significant for understanding the characteristics of ST evolution and land‒atmosphere interactions on the Qinghai-Tibet Plateau and provides important data support for improving the underlying surface boundary conditions of models.

## Introduction

Global temperature has increased rapidly in recent decades. Climate change, as a hot issue discussed in today's society, has attracted much attention. The Intergovernmental Panel on Climate Change (IPCC) Fifth Assessment Report (AR5) pointed out that from 1980 to 2012, which may have been the warmest 30 years in the Northern Hemisphere over the past 1400 years, multiple global land and ocean reanalysis datasets show an average global warming of 0.85 °C [0.65 °C to 1.06 °C] from 1880 to 2012^1,2^. The Qinghai-Tibet Plateau (QTP) is located in the most active region of global climate change and is the initiator and regulator of climate change in the Northern Hemisphere^3,4^. Many studies have shown that the QTP is one of the most sensitive regions to global climate change, with characteristics of sensitivity, advancement and regulation^5,6^. Climate change on the plateau not only directly drives climate change in eastern and southwestern China but also affects East Asia, the Northern Hemisphere, and even the whole world^7^.

The Qinghai-Tibet Plateau is located in western China and includes Tibet, Qinghai, southern Xinjiang, western Sichuan, southern Gansu, and northwestern Yunnan. The QTP spans more than 2500 km from east to west and is approximately 1000 km from north to south. The total area is nearly $2.5 \times 10^{6} {\text{km}}^{2}$, accounting for approximately one-fourth of China's total area. It has an average elevation of 4000 m, which accounts for almost one-third of the thickness of the troposphere. The QTP, often referred to as the *Roof of the World* or *Third Pole*, is known for its complex terrain and high altitude^8^. The QTP plays an important role in climate change and land‒atmosphere interactions^9–11^, and its climate and ecological environment are jointly affected by westerly winds and Asian monsoons. In turn, the Qinghai-Tibet Plateau has also affected regional and global climate change^12^. For example, the snow cover in winter and spring on the Qinghai-Tibet Plateau has a profound impact on temperature and precipitation in China and even in Asia^13,14^. Changes in summer heat sources on the Qinghai-Tibet Plateau have a key impact on the evolution of the Asian monsoon^15–17^.

However, the Qinghai-Tibet Plateau, as a giant tectonic landform with the highest altitude in the world, has a unique natural environment as well as spatial distribution, and is restricted by atmospheric circulation and plateau topography, thus forming unique hydrothermal conditions^18^. The fundamental driving force of atmospheric motion is solar radiation, which then heats the atmosphere through the underlying surface. Therefore, changes in the thermal properties of the underlying surface govern the formation and changes in atmospheric circulation. As an important part of the underlying surface of the land, soil is the lower boundary of material and energy exchange in the earth-atmosphere system^19^. Soil temperature (ST) is one of the important parameters used to characterize the thermal properties of soil, and it plays an important role in the research of many related fields^20^. In terms of the energy cycle, soil temperature affects climate change by affecting changes in surface energy. Therefore, the diagnosis and prediction of ST are important scientific and technical issues in land-surface process models, numerical weather prediction and short-term climate prediction^21^. In terms of the water cycle, ST greatly affects the processes of sensible heat, latent heat and surface evapotranspiration. Changes in ST can further affect the temperature and precipitation of the Qinghai-Tibet Plateau and East Asia through the exchange of water between the atmosphere and the surface^22,23^. In agricultural research, soil temperature is a driving environmental factor that affects crop growth, fertilizer decomposition and organic matter accumulation^24,25^. Considering meteorological research, the change in ST is a reliable indicator reflecting climate change. For example, the increase in ST in permafrost regions is an important indicator of permafrost degradation^26–28^. The shallow ST is also one of the key factors of precipitation. Because the heat in the shallow soil can be easily released into the atmosphere, the abnormality of the shallow ST will affect the short-term weather process^29^.

Because of the importance of ST, many scholars have done much work. Tang et al.^30^ conducted a series of studies on the relationship between ST and precipitation and found a good corresponding relationship between ST and later precipitation, and the ST at different levels had different continuity. The work of Qin et al.^31^ showed that the increasing trend of shallow ST in the Qinghai-Tibet Plateau in spring and summer was greater than that of deep soil; the temperature increase rate of shallow soil in autumn and winter was significantly lower than that of deep soil; the significant positive growth trend of the annual average ST indicated that the Qinghai-Tibet Plateau has responded to climate warming in the past few decades, and it is also regarded as one of the important indicators of permafrost degradation on the Qinghai-Tibet Plateau. Studies have shown that ST is one of the key factors affecting soil respiration in ecosystems such as forests, grasslands, and farmland^32–34^. The sensitivity of the soil respiration rate to changes in ST is also an important link in the carbon cycle of terrestrial ecosystems^35^. Considering the different effects of ST on climate and ecology, scholars have also conducted regional analyses of ST. Due to the synergy of many factors, such as solar radiation, atmospheric circulation, and precipitation, there is a complex energy exchange between the soil and atmosphere. This forms periodic diurnal and seasonal changes in soil temperature and forms different regional characteristics of soil temperature^36^. Gao et al.^37^ used fuzzy C-means clustering to classify ST at 1.6 m in China into four categories: the cold region ($\overline{T}_{1.6} \le 9\;^\circ {\text{C}}$), including Northeast China and the Qinghai-Tibet Plateau; the subcold region ($9\;^\circ {\text{C}} \le \overline{T}_{1.6} \le 15{ }\;^\circ {\text{C}}$), mainly including Northwest China; the subwarm region ($15\;^\circ {\text{C}} \le \overline{T}_{1.6} \le 18\;^\circ {\text{C}}$), including the Huaihe Basin area; and the warm region ($\overline{T}_{1.6} \ge 18\;^\circ {\text{C}}$), including southern China. Zhou et al.^38^ used the fuzzy C-means clustering method to classify ST at 3.2 m depth from 113 sites in China and divided them into 10 regions. The research showed that there were obvious regional differences in the increasing rate of the 3.2 m ST. Northeast and Northwest China had the largest temperature increases, approximately 0.57 K∕10a, and the other regions were all below 0.36 K∕10a.

As one of the most sensitive areas of global climate change, the Qinghai-Tibet Plateau is an indispensable part of understanding the degradation of frozen soil, the ecological environment and climate change. Research^39^ has shown that due to the increase in soil temperature in the past 30 years, the permafrost layer with a temperature higher than − 2 °C has rapidly degraded, and in some places (such as the North Jingxian Valley), the permafrost layer has become even thinner by 5 m within 15 years. Because of the importance of soil temperature on the Qinghai-Tibet Plateau to the study of atmospheric circulation^40–43^, hydrology^44,45^, and permafrost^31^, it is necessary to accurately describe the characteristics of soil temperature. To date, most studies on ST in the Qinghai-Tibet Plateau are been based on model results or reanalysis data from different sources. However, due to the harsh environmental conditions and the complex topography of the Qinghai-Tibet Plateau, the model parameters of the QTP are not sufficiently accurate. The soil temperature data obtained from the model have limitations and uncertainties^46,47^. The reanalysis data from different sources, because of the use of different assimilation systems, are also different from each other, and there is a relatively large error with the observations. For example, research^31^ has shown that the European Centre for Medium-Range Weather Forecasts interim reanalysis (ERA-Interim), the second version of the National Centers for Environmental Prediction Climate Forecast System (CFSv2), and the Modern-Era Retrospective Analysis for Research and Applications, version 2 (MERRA-2), all have significant cold bias. The reason for this kind of cold bias may be that the surface models used in the reanalysis products were not originally developed for high latitudes and complex terrain regions. Therefore, these models do not adequately describe the unique hydrothermal conditions of the underlying surface of the Qinghai-Tibet Plateau represented by permafrost regions^48–50^. In terms of the use of observational data, most studies have focused on the time-varying characteristics of some observation sites. For example, Wan et al.^51^ analyzed the air temperature and soil temperature data of six observation stations along the Qinghai-Tibet Plateau and found that the soil temperature increase trend in winter was significantly greater than that in summer, and the daily minimum soil temperature increase rate was greater than the daily maximum soil temperature. Soil temperature showed a clear response to climate warming, but the response varied significantly with depth and location. Zhao et al.^6^ used the soil temperature observation data from 74 sites from 1977 to 2006 to study the soil thermal conditions of the Qinghai-Tibet Plateau. The results showed that the absolute value of the 5 cm soil negative accumulated temperature had a decreasing trend, and the decrease range was different in different regions.

In view of the fact that most of the reanalysis data have cold biases and most of the observational data are located in the eastern part of the Qinghai-Tibet Plateau, with fewer stations in the western part^6^, this work was based on the observation data from 141 stations on the QTP, using the “Moving Surface Spline Interpolation Algorithm Based on Green’s Function” to interpolate the data of the observation site and develop a set of grid data. It has a long time span (1981–2020) and a relatively high spatial resolution ($0.25^\circ \times 0.25^\circ$) and verifies the accuracy of this set of grid data (the errors in most regions do not exceed 3%). This work used these grid data to analyze the temporal and spatial variation characteristics of soil temperature in different layers of the Qinghai-Tibet Plateau and divided the shallow soil temperature of the Qinghai-Tibet Plateau into 7 regions. This work is significant for understanding the characteristics of ST evolution and land‒atmosphere interactions on the Qinghai-Tibet Plateau and provides important data support for improving the underlying surface boundary conditions of land-surface process models.

## Data and methods

### Data selection

The observational data in this work were the daily ST of 141 stations on the Qinghai-Tibet Plateau from January 1, 1981, to December 31, 2020, by the National Climate Centre. The dataset has nine soil layers with depths of 0, 5, 10, 15, 20, 40, 80, 160, and 320 cm. In the northwestern part of the Qinghai-Tibet Plateau, there are fewer ST observation sites. This work analyzed the soil temperature at 0, 5, 10, 15, 20, and 40 cm, that is, the shallow soil temperature.

**Figure 1:**
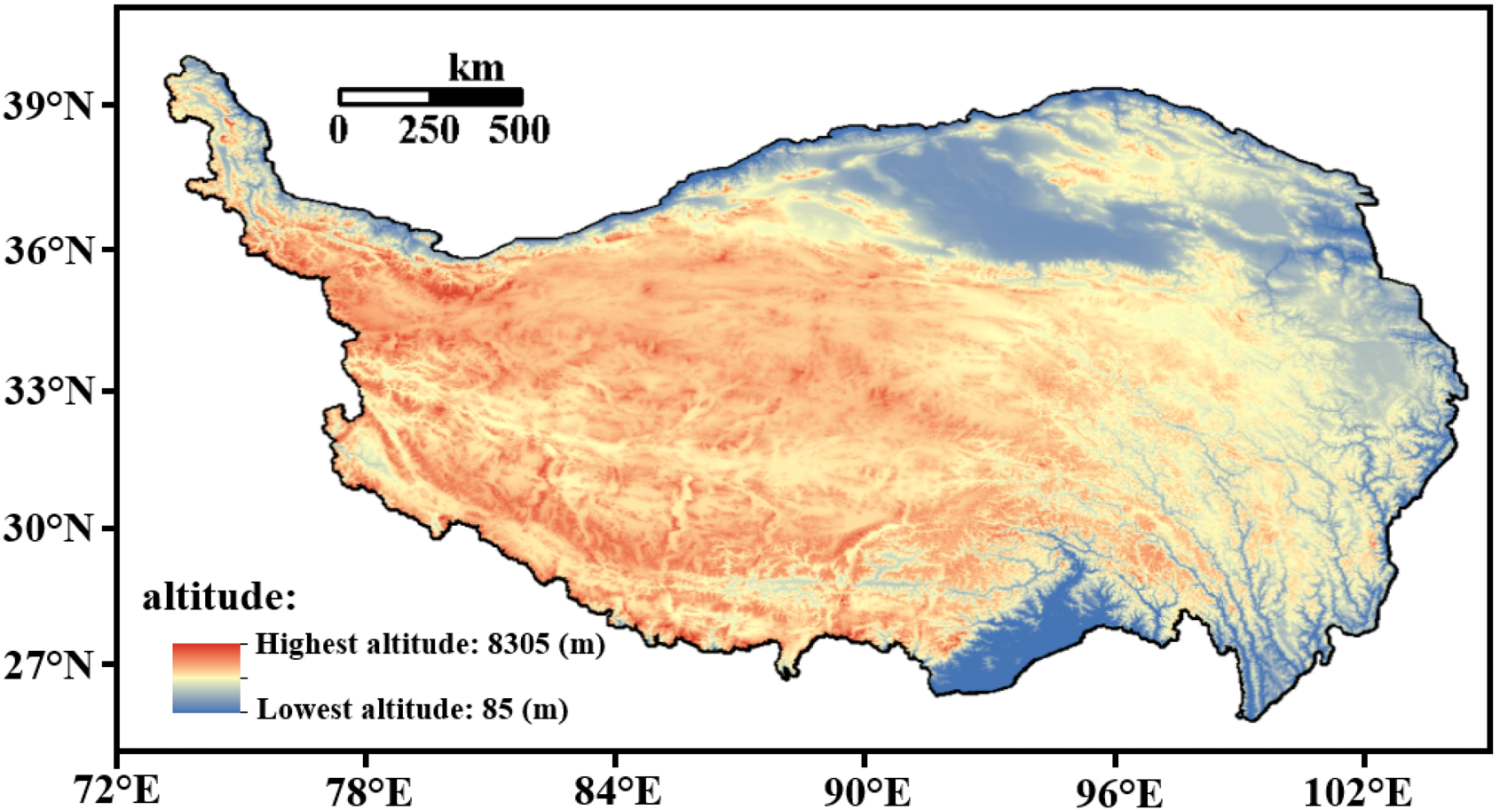
The altitude of the Qinghai-Tibet Plateau. (Software: ArcGIS 10.5, https://www.arcgis.com/index.html).

**Figure 2:**
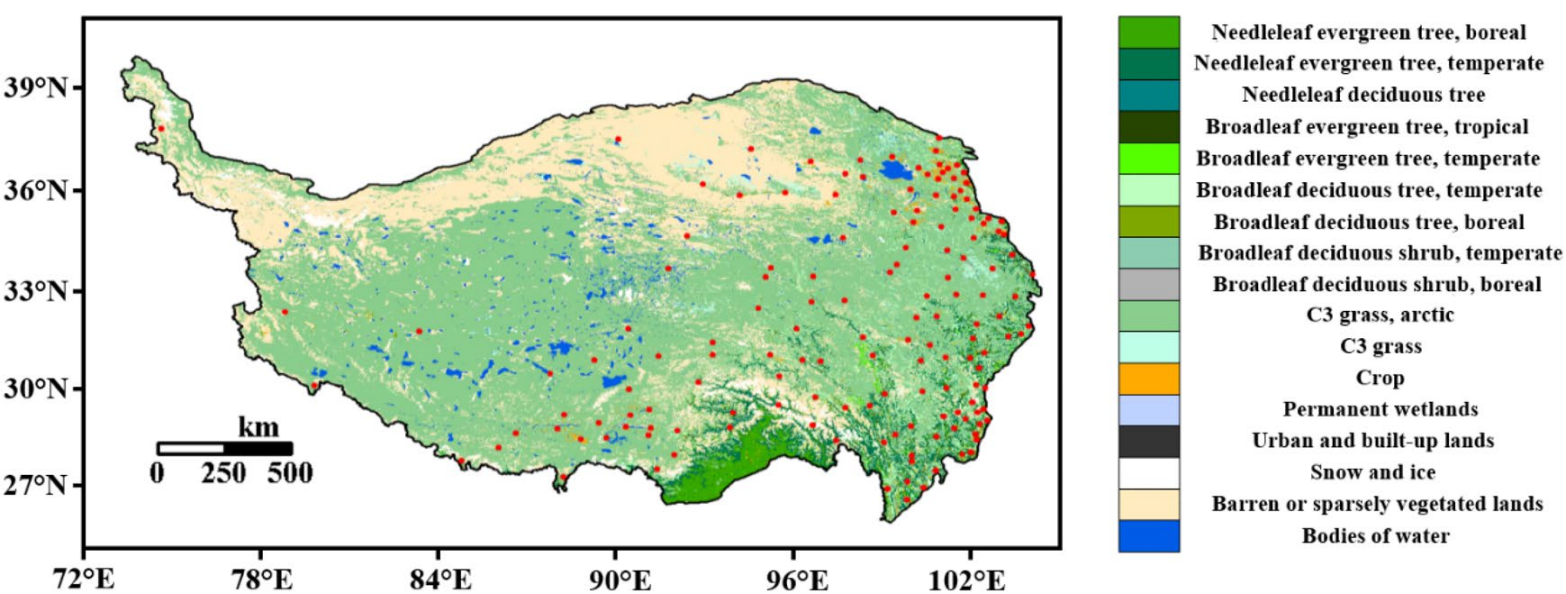
The underlying surface types and observation sites of the Qinghai-Tibet Plateau. (The data source of the land cover map comes from Ran et al.^52^, Software: ArcGIS 10.5, https://www.arcgis.com/index.html).

Considering the unique geographical conditions and underlying surface types of the Qinghai-Tibet Plateau, the altitude, underlying surface types and observation sites of the Qinghai-Tibet Plateau are shown in Figs. 1 and 2.

Reanalysis data are widely used for documenting and interpreting persistent changes within the Qinghai-Tibet Plateau^53^. Different reanalysis data differ in various aspects, such as numerical prediction models, assimilation schemes, and data sources^54^. You et al.^55^ showed that the temperature provided by the European Centre for Medium-Range Weather Forecasts (ECMWF) over the Qinghai-Tibet Plateau was better than that from the NCEP. ECMWF produced the ERA5 reanalysis data in September 2019, which has higher spatial and temporal resolution, improved surface parameterization and cloud precipitation models, and added more historical observation data to the assimilation system^54^. In particular, satellite data are used to assimilate systems and model systems^56^. The ST data we used were in the second layer ERA5 hourly data on single levels from 1979 to the present (copernicus.eu). The ECMWF Integrated Forecasting System (IFS) has a four-layer representation of soil, where the surface is at 0 cm: Layer 1: 0–7 cm, Layer 2: 7–28 cm, Layer 3: 28–100 cm, and Layer 4: 100–289 cm. The second layer was chosen in this study, ST was actually set in the middle of each layer, and the second layer was 18.5 cm. Since the temporal and spatial changes in the ST at different layers of shallow soil have high similarity^57^, this study compared the errors between the second layer (18.5 cm) of the reanalysis data and the observation data at the nearest layer (20 cm).

### Fuzzy C-means algorithm (FCM)

In this paper, the fuzzy C-means algorithm (FCM) was used to classify the annual average soil temperature of 141 ST observation sites at 20 cm on the Qinghai-Tibet Plateau from 1981 to 2020. Clustering is a method that divides a collection of abstract objects into different classes or clusters according to a certain standard (such as distance criterion, that is, the distance between different data), so that the similarity of data objects in the same cluster and the difference between different clusters is as great as possible^58^. The main idea of the FCM is to divide a number of L-dimensional vectors into C fuzzy groups, determine the degree of membership of each category by distance, and continuously update the membership degree as well as the clustering center of image pixels to minimize the objective function and complete pixel classification and image segmentation^59^. The pixel membership degree is used to describe the degree to which the pixel belongs to a certain category, and the value range is [0,1]. The objective function and restriction of the FCM algorithm are as follows:

1 $$J_{FCM} = \mathop \sum \limits_{i = 1}^{C} \mathop \sum \limits_{j = 1}^{N} u_{ij}^{m} d_{ij}^{2} \left( {x_{j} ,v_{i} } \right), \;\;\;\;\mathop \sum \limits_{i = 1}^{C} u_{ij} = 1$$

where $u_{ij} = u_{j} \left( {x_{j} } \right)$ represents the degree to which pixel gray $x_{j}$ belongs to category $i$; $m$ is the fuzzy weighting coefficient, often taken as 2; $v_{i} = \left\{ {v_{1} , \ldots ,v_{c} } \right\}$ represents the $i - th$ cluster centers; and $d_{ij} \left( {x_{j} ,v_{i} } \right) = ||x_{j} - v_{i} ||^{2}$ represents the shortest distance from the gray level of the $j - th$ pixel to the $i - th$ cluster center.

Using the Lagrangian multiplier method to find the minimum value of the objective function,

2 $$J_{\lambda } = \mathop \sum \limits_{i = 1}^{C} \mathop \sum \limits_{j = 1}^{N} u_{ij}^{m} d_{ij}^{2} \left( {x_{j} ,v_{i} } \right) + \lambda \left( {\mathop \sum \limits_{j = 1}^{C} u_{ij} - 1} \right)$$

3 $$v_{i} = \frac{{\mathop \sum \nolimits_{j = 1}^{N} u_{ij}^{m} x_{j} }}{{\mathop \sum \nolimits_{j = 1}^{N} u_{ij}^{m} }} \left( {i = 1, \ldots ,C} \right)$$

4 $$u_{ij} = \frac{1}{{\mathop \sum \nolimits_{k = 1}^{N} \left( {\frac{{d\left( {x_{j} ,v_{i} } \right)}}{{d\left( {x_{j} ,v_{k} } \right)}}} \right)^{{2\left( {m - 1} \right)}} }}$$

The standard FCM algorithm is superior in simple image segmentation. In recent years, FCM has been widely used in research fields such as climate region division, pattern recognition, data analysis and image processing, showing that FCM can clearly represent the continuous spatial distribution of natural phenomena^60^.

### Mann–Kendall mutation analysis

The change in ST is synergistically affected by many factors, so it can show the characteristics of trend change. The Mann–Kendall (M–K)^61,62^ test is not affected by the sample value, distribution type, etc., and further analyzes the change of variables by deeply mining the hidden information within the time series. Therefore, it is widely used in the research of time analysis of natural variables. MK mutation analysis is a nonparametric statistical test method that is not only easy to calculate but can also identify the time when the mutation starts and point out the region of the mutation time period.

Given the time series variables $\left( {X_{1} ,X_{2} , \ldots ,X_{n} } \right)$, where $n$ is the length of $X$, the rank of the series is defined as follows:

5 $$s_{k} = \mathop \sum \limits_{i = 1}^{k} r_{i}$$

where $r_{i}$ can be expressed as follows:

6 $$r_{i} = \left\{ {\begin{array}{*{20}c} { + 1, if x_{i} > x_{j} } \\ {0, else} \\ \end{array} } \right.$$

It can be seen that $s_{k}$ is the accumulation of the number of values at the $i - th$ time greater than the ${ }j - th$ time.

Under the assumption of random independence of the time series, define the statistics:

7 $$UF_{k} = \frac{{\left[ {s_{k} - E\left( {s_{k} } \right)} \right]}}{{\sqrt {Var\left( {s_{k} } \right)} }} \left( {k = 1,2, \cdots ,n} \right)$$

where $UF_{1} = 0$, $E\left( {s_{k} } \right)$ and $Var\left( {s_{k} } \right)$ are the mean and variance of the accumulated number $s_{k}$. When $X_{1} ,X_{2} , \ldots ,X_{n}$ are independent of each other and have the same continuous distribution, then,

$$E\left( {s_{k} } \right) = \frac{{n\left( {n - 1} \right)}}{4},\;Var\left( {s_{k} } \right) = \frac{{n\left( {n - 1} \right)\left( {2n + 5} \right)}}{72}$$

where $UF_{k}$ is a standard normal distribution, which is a series of statistics calculated in the order of time series $X_{1} ,X_{2} , \ldots ,X_{n}$. Given significance level ${\upalpha }$, if $\left| {UF_{k} } \right| > U_{\alpha }$, the series has obvious trend changes.

The time series is generated into its corresponding inverse sequence $X_{n} ,X_{n - 1} , \ldots ,X_{1}$, and the above calculation process is repeated. Meanwhile, $UB_{k} = - UF_{k}$, ${\text{k}} = {\text{n}},{\text{n}} - 1, \ldots ,1,{ }UB_{1} = 0$.

To summarize the brief computational steps for Mann–Kendall (M–K) mutation analysis:

1. Calculate the rank column of the sequential time series, and calculate $UF_{k}$ according to the rank;
2. Calculate the rank column of the reverse time series, and calculate $UB_{k}$ according to the rank;
3. Given a significance level, such as ${\upalpha } = 0.05$, for the critical value $U_{0.05} = \pm 1.96$, if $UF$ or $UB$ is greater than 0, it indicates that the sequence is in an upward trend; otherwise, it is in a downward trend. If it exceeds the critical value, the upward or downward trend is significant; if there is an intersection between $UF$ and $UB$, and the intersection is between the critical lines, the intersection is the moment when the mutation begins.

### Moving surface spline interpolation algorithm based on Green’s function

 The data of 141 ST observation stations on the Qinghai-Tibet Plateau were interpolated by *the moving surface spline interpolation algorithm based on Green’s function*. Compared with Shepard (IDW interpolation), bivariate cubic polynomial fitting, local neighborhood kriging, ordinary kriging, moving surface spline interpolation, etc., this method has higher accuracy and better continuity when dealing with complex terrain and different underlying surface conditions^63^.

The solution of Green’s function implies that the surface $s\left( x \right)$ can be expressed as follows:

8 $$s\left( x \right) = T\left( x \right) + \mathop \sum \limits_{j = 1}^{n} w_{j} g\left( {x,x_{j} } \right)$$

where $x$ is the output position vector of the unknown data point, $g\left( {x,x_{j} } \right)$ is Green's function, $x_{j}$ is the j-th data constraint, and $w_{j}$ is the associated unknown weight relative to $x$. $T\left( x \right)$ is the trend function^64^. The weight $w_{j}$ is determined by requiring Eq. (8) to be accurately satisfied in n data positions:

9 $$s\left( {x_{i} } \right) = \mathop \sum \limits_{j = 1}^{n} w_{j} g\left( {x_{i} ,x_{j} } \right), i = 1,2, \ldots ,n.$$

10 $$g\left( {x_{i} ,x_{j} } \right) = |x_{i} ,x_{j} |^{2} \ln \left( {\left| {x_{i} ,x_{j} } \right|} \right)$$

The steps of the moving surface spline interpolation algorithm based on Green's function are as follows:

Part (1). Assuming a surface has a total of n known points, the desired output node is $p_{0}$*.* The number of points nearest to $p_{0}$ used for interpolation is *k*. The distance from point $p_{0}$ to other known points should be calculated first as follows:

11 $$r_{0i} = \left| {x_{i} ,x_{0} } \right|, i = 1,2, \ldots ,n$$

where $r_{0i}$ is the distance between point $p_{0}$ and the $i - th$ known data point,$x_{0}$ is the position vector of $p_{0}$, and $x_{i}$ is the position vector of the $i - th$ known data point.

Part (2). Setting the coordinate matrix $X = \left[ {x_{1} x_{2} \ldots x_{k} } \right]^{T}$, $Y = \left[ {y_{1} y_{2} \ldots y_{k} } \right]^{T}$, attribute matrix $Z = \left[ {z_{1} z_{2} \ldots z_{k} } \right]^{T}$, $k \times k$ Green’s function matrix $G$ is as follows:

12 $$G = \left[ {\begin{array}{*{20}l} {\begin{array}{*{20}c} {d_{11} } & {d_{12} } \\ {d_{21} } & {d_{22} } \\ \end{array} } \hfill & {\begin{array}{*{20}c} \cdots & {d_{1k} } \\ \cdots & {d_{2k} } \\ \end{array} } \hfill \\ {\begin{array}{*{20}c} \vdots & \vdots \\ {d_{k1} } & {d_{k2} } \\ \end{array} } \hfill & {\begin{array}{*{20}c} \ddots & \vdots \\ \cdots & {d_{kk} } \\ \end{array} } \hfill \\ \end{array} } \right]$$

13 $$d_{ij} = \left\{ {\begin{array}{*{20}c} {\begin{array}{*{20}c} 0 \\ {\left[ {\ln \left( {r_{ij} } \right) - 1.0} \right]r_{ij}^{2} } \\ \end{array} } & {\begin{array}{*{20}c} {r_{ij} = 0} \\ {r_{ij} \ne 0} \\ \end{array} } \\ \end{array} } \right. i = 1, \cdots ,k,j = 1, \cdots ,k$$

where $r_{ij} = \sqrt {\left( {x_{i} - x_{j} } \right)^{2} + \left( {y_{i} - y_{j} } \right)^{2} }$. The weight matrix *W* can be calculated as follows:

14 $$W = G^{ - 1} Z$$

Part (3). Computing *1* × *k* Green’s function matrix $G_{p}$ of point $p_{0}$:

15 $$G_{p} = \left[ {d_{01} d_{02} \cdots d_{0k} } \right]$$

where $d_{01} ,d_{02} , \cdots ,d_{0k}$ are calculated by Eq. (13). The attribute value of point $p_{0}$ is as follows:

16 $$z_{p} = G_{p} W$$

Part (4). Repeat Parts (1)–(3) to calculate the interpolation $z_{pi}$ of other points $p_{i}$.

### Estimating the interpolation error

Interpolation error $e_{{z_{p} }}$ is related to the interpolation result $p$ and attribute value of the known points. Interpolation error $e_{{z_{p} }}$ is defined as follows:

17 $$e_{{z_{p} }} = \hat{z}_{p} - z_{p}$$

where $\hat{z}_{p}$ is the true attribute value of point $p$ and $z_{p}$ is the interpolation value. However, Eq. (17) has only theoretical meaning because $\hat{z}_{p}$ cannot be obtained. Therefore, $e_{{z_{p} }}$ should be calculated by other methods.

From Eq. (16), the derivation of $z_{p}$ is as follows:

18 $$dz_{p} = G_{p} dW$$

From Eq. (14), the derivation of $W$ is as follows:

19 $$dW = G^{ - 1} dZ$$

Combining Eqs. (18) with (19),

20 $$dz_{p} = G_{p} G^{ - 1} dZ$$

According to the error propagation law,

21 $$e_{{z_{p} }} = \left[ {\left( {G_{p} G^{ - 1} } \right)\left( {G_{p} G^{ - 1} } \right)^{T} } \right]^{\frac{1}{2}} m_{z}$$

where $m_{z}$ is the mean square error of the attribute value $Z$. In practical applications, matrix $G$ is a fixed constant matrix and relates only to the location of known points. For output point $p_{0} \left( {x_{0} ,y_{0} } \right)$, the elements of matrix $G_{p}$ are calculated from the distances between point $p_{0}$ and other points^63^.

## Results

### Temporal and spatial distribution of shallow layer STs on the Qinghai-Tibet Plateau

#### The spatial distribution

**Figure 3:**
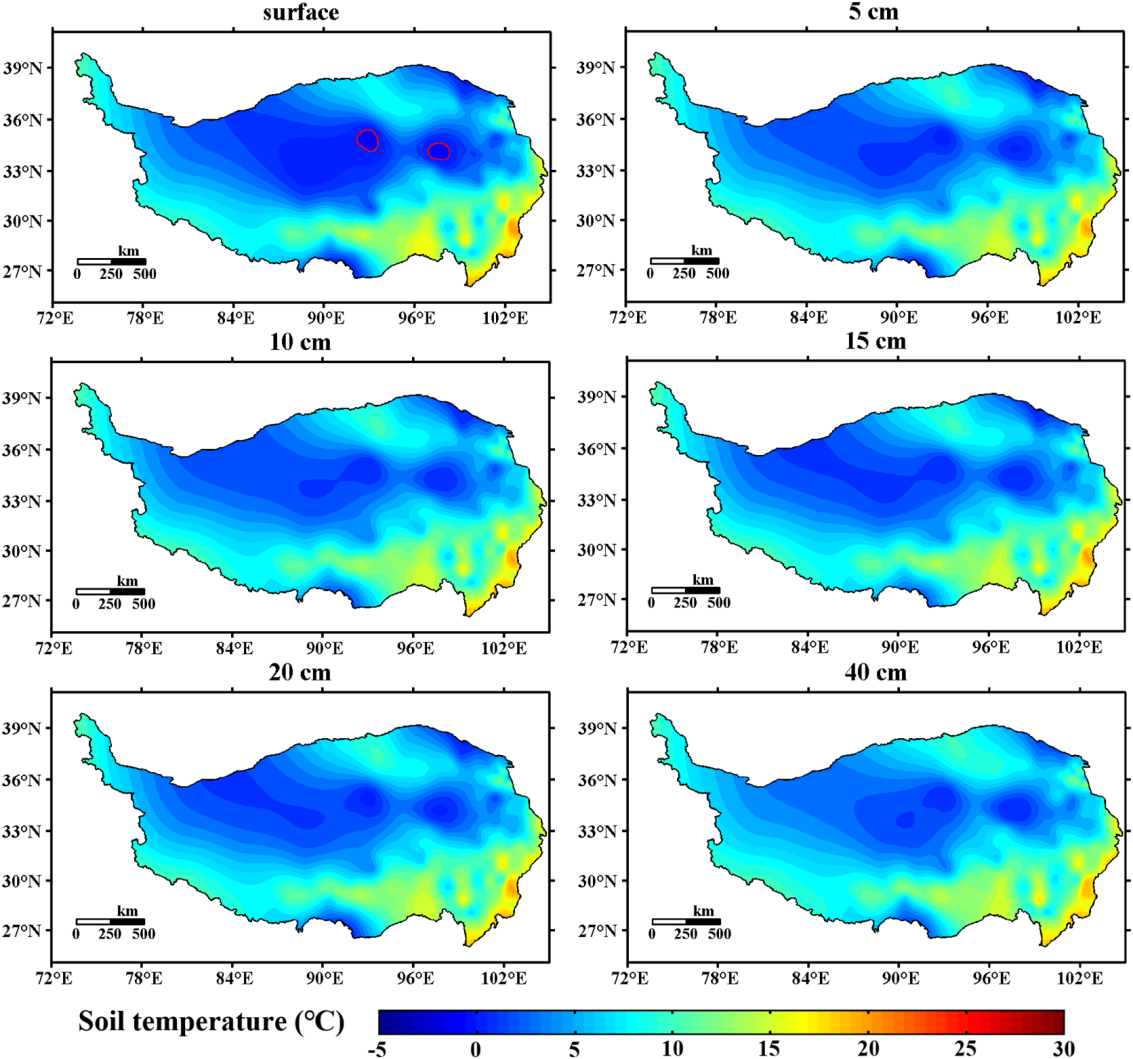
Spatial distribution of annual average ST in the shallow layer of the Qinghai-Tibet Plateau from 1981 to 2020 (unit:  °C, Software: MATLAB 2014, https://ww2.mathworks.cn/products/matlab.html).

Figure 3 shows the spatial distribution of the annual average ST in the shallow layer of the Qinghai-Tibet Plateau from 1981 to 2020 (0 cm, 5 cm, 10 cm, 15 cm, 20 cm, and 40 cm). It can be seen from the figure that the shallow ST of the Qinghai-Tibet Plateau has a gradual increasing trend from north to south, but there is a relatively high ST area in Golmud, Qinghai, with an average of 5–10 °C. In the vast areas of the Northern Qiangtang Plateau and the Southern Qiangtang Plateau, the shallow ST is basically below 5 °C. The soil temperature in southeastern Tibet and the western Sichuan Plateau is high, reaching above 20 °C.

#### Temporal characteristics

**Figure 4:**
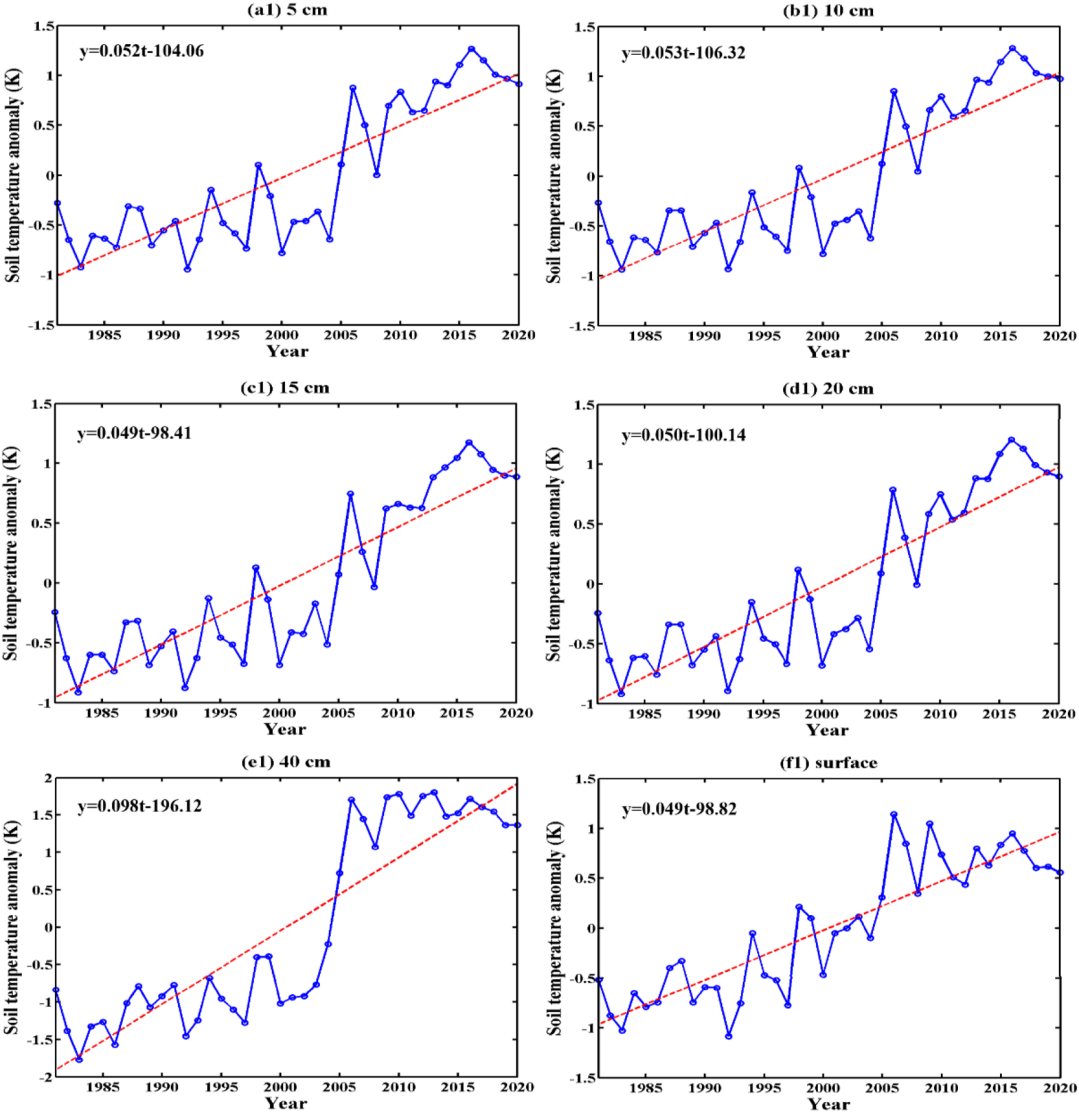
Interannual variation in shallow ST anomalies on the Qinghai-Tibet Plateau (unit: K, ${\upalpha } = 0.01$).

**Figure 5:**
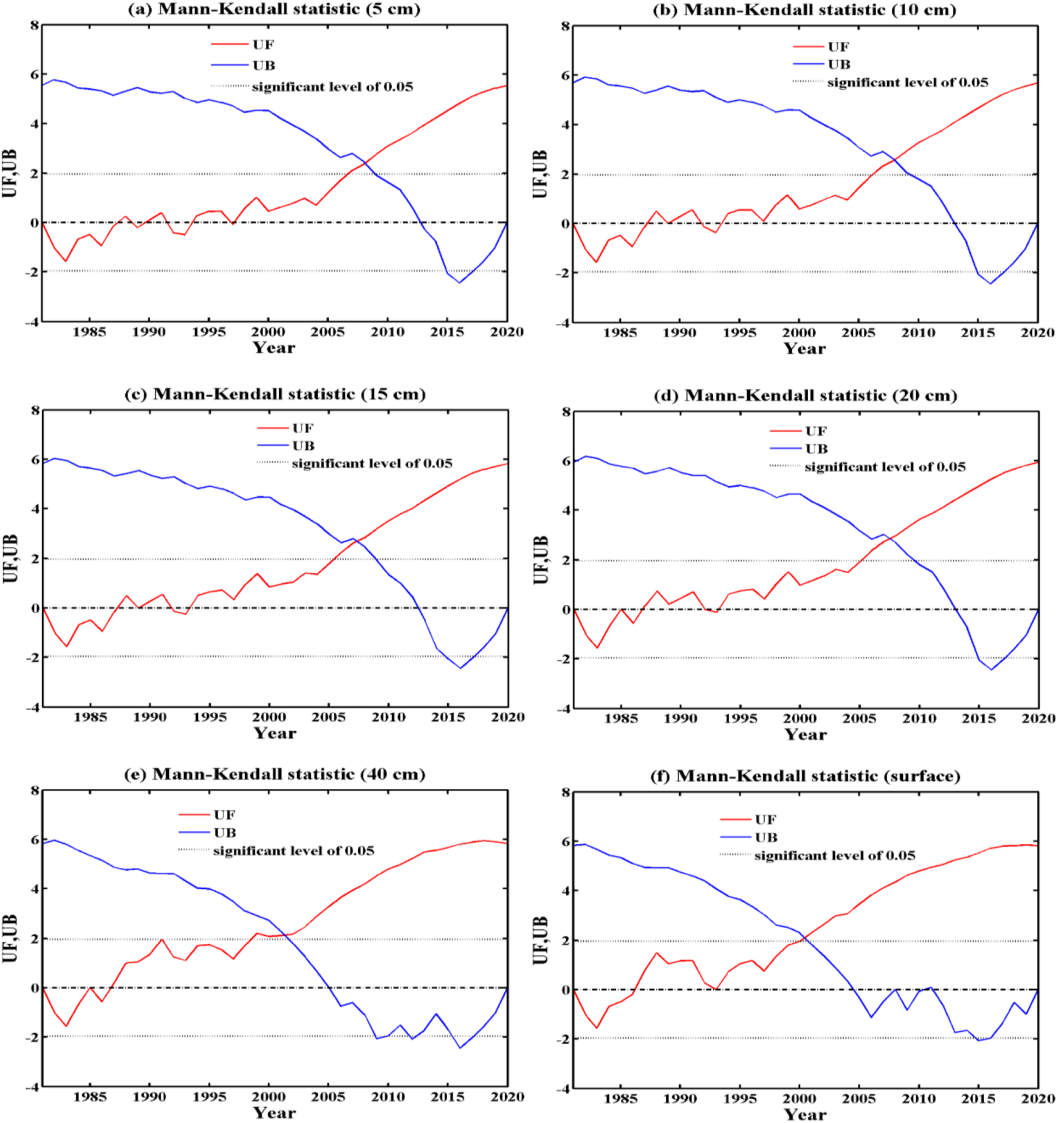
MK analysis of shallow ST on the Qinghai-Tibet Plateau. $UF$ and $UB$ are the statistics of the MK analysis.

Figures 4 and 5 show the interannual variation in ST anomalies in the shallow layer (0 cm, 5 cm, 10 cm, 15 cm, 20 cm, and 40 cm) of the Qinghai-Tibet Plateau and their MK tests. From 1981 to 2020, the ST at the six levels showed a significant upward trend, but the rising amplitudes of each level were quite different. From the MK tests in Fig. 5, it can be seen that 5 cm, 10 cm, 15 cm, and 20 cm had insignificant mutation points near 2008. In addition, 40 cm and 0 cm had mutations near 2002. Table 1 shows the statistical information of the annual and seasonal temperatures of the shallow soil on the Qinghai-Tibet Plateau, including the average, standard deviation, and interdecadal variability. On an annual scale, the average annual ST of 0–20 cm was 9.15–9.57 °C, and that of 40 cm was 8.69 °C. The interdecadal variability of 0–20 cm was 0.49–0.53 K/10a, and the interdecadal variability of 40 cm reached 0.98 K/10a, which was much higher than that of the other levels. From the seasonal perspective, the interdecadal variability in ST in winter and spring was relatively high, with 40 cm even reaching more than 1 K/10a. In addition, the 40 cm interannual and seasonal ST standard deviations (1.13–1.44 K) were much larger than those of the other layers (σ < 1 K), indicating that the ST at 40 cm varied more greatly. This result was reflected in the mutation point in 2002–2006 of Fig. 4(e1) and in 2002 of Fig. 5(e). The analysis shows that the temperature of the shallow soil on the Qinghai-Tibet Plateau has had a clear increasing trend. The temperature increase of 0–20 cm (the surface layer of shallow soil) was roughly the same, which was quite different from that of 40 cm (the bottom layer of shallow soil).

**Table 1:** Statistical information on the annual average and interannual variations in various seasons on the Qinghai-Tibet Plateau from 1981 to 2020.

Layer	Season	Statistical information		
		μ (°C)	σ (K)	Trend (K/10a)
5 cm	Spring	10.19	0.78	0.55**
	Summer	18.42	0.65	0.39**
	Autumn	9.42	0.77	0.51**
	Winter	− 1.43	0.93	0.63**
	Annual	9.15	0.71	0.52**
10 cm	Spring	9.74	0.85	0.61**
	Summer	18.15	0.70	0.45**
	Autumn	9.81	0.75	0.50**
	Winter	− 0.96	0.83	0.55**
	Annual	9.19	0.72	0.53**
15 cm	Spring	9.27	0.74	0.48**
	Summer	17.87	0.63	0.37**
	Autumn	10.28	0.71	0.49**
	Winter	− 0.30	0.70	0.62**
	Annual	9.28	0.66	0.49**
20 cm	Spring	9.05	0.83	0.60**
	Summer	17.62	0.67	0.45**
	Autumn	10.41	0.68	0.46**
	Winter	− 0.17	0.71	0.48**
	Annual	9.22	0.67	0.50**
40 cm	Spring	7.65	1.51	1.14**
	Summer	16.46	1.13	0.84**
	Autumn	10.58	1.17	0.87**
	Winter	0.06	1.44	1.06**
	Annual	8.69	1.29	0.98**
surface	Spring	11.17	0.75	0.51**
	Summer	19.51	0.61	0.30**
	Autumn	9.38	0.75	0.51**
	Winter	− 1.79	0.96	0.66**
	Annual	9.57	0.66	0.49**

**μ** mean, **σ** standard deviation.

**Denotes trends statistically significant at α = 0.001.

#### Characteristics of spatial changes in shallow soil layers of the Qinghai-Tibet Plateau

**Figure 6:**
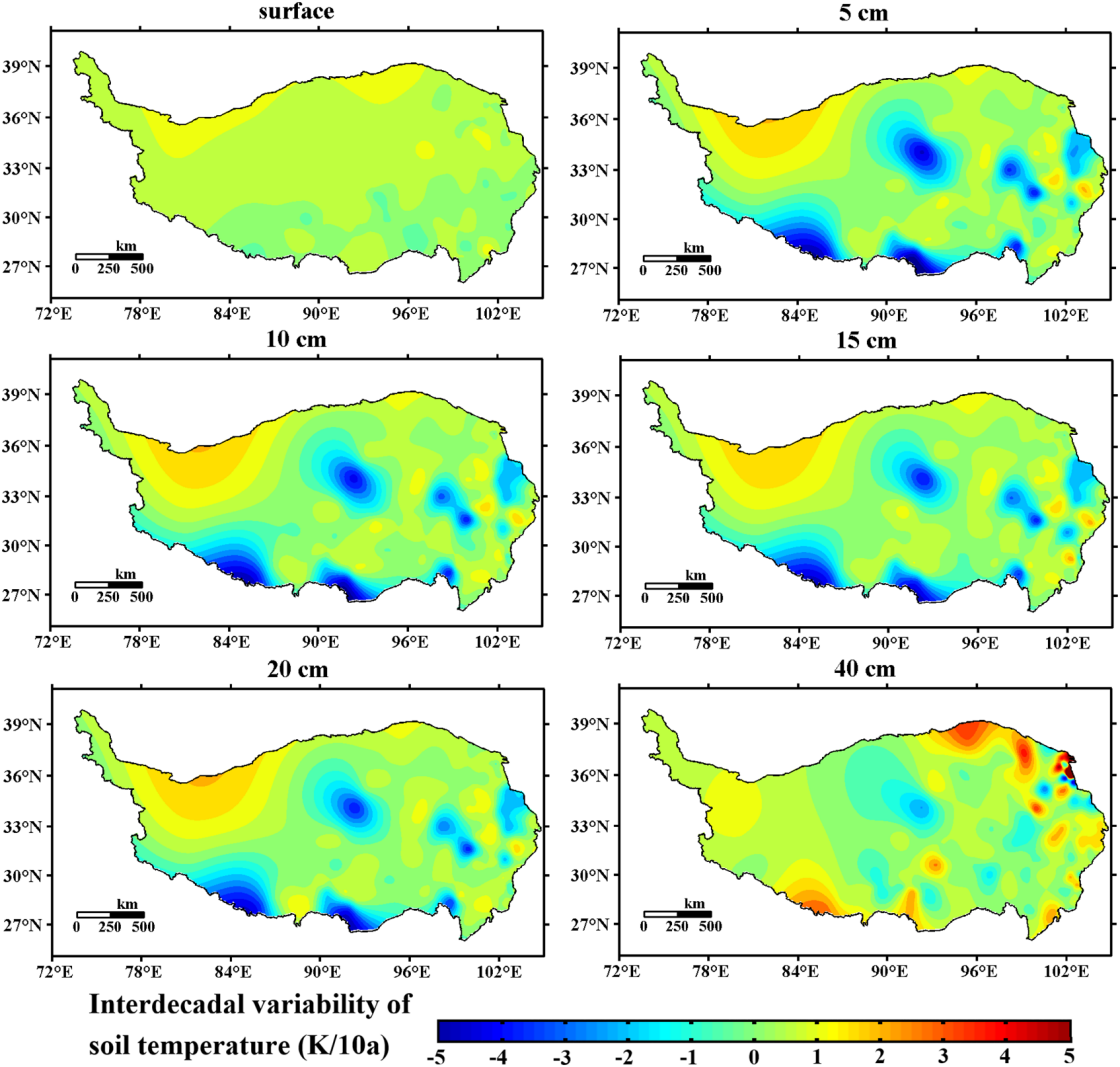
Distribution of interdecadal variability in ST in shallow soil on the Qinghai-Tibet Plateau from 1981 to 2020 (unit: K/10 a, ${\upalpha } = 0.01$, Software: MATLAB 2014, https://ww2.mathworks.cn/products/matlab.html).

Figure 6 shows the interdecadal distribution of ST in shallow layers (0 cm, 5 cm, 10 cm, 15 cm, 20 cm, and 40 cm) of the 141 observation sites on the Qinghai-Tibet Plateau from 1981 to 2020. The results are significant at the 99% (${\upalpha } = 0.01$) confidence level. As shown in the figure, the interdecadal variabilities of 5–20 cm (the surface layer of the shallow soil) are basically the same. In central Tibet and the southern and southwestern parts of the Qinghai-Tibet Plateau, the ST had a significant cooling trend, and the interdecadal variabilities were as low as − 1 K/10a. Li et al.^65^ pointed out that since the beginning of the twenty-first century, in the central and southwestern regions of the Qinghai-Tibet Plateau, the surface heat source has been decreasing with a trend of $- 0.2{\text{ W}}m^{ - 2} decadal^{ - 1}$. Li et al.^66^ found that since 2006, the Qinghai-Tibet Plateau has generally maintained a warming trend, but some regions, especially the central and southern parts of the plateau, have experienced slower or even “stagnant” trends. These may be partly responsible for the ST cooling trend in the central, southern and southwestern regions of the Tibetan Plateau. In the northwestern part of the Qinghai-Tibet Plateau, the ST is obviously warming. Compared with the other layers, 40 cm (the bottom layer of shallow soil) is quite different. Overall, the temperature increase trend at 40 cm was more obvious than that of the other layers. The cooling trend still exists in central Tibet; the warming trend in the northwestern Qinghai-Tibet Plateau has slightly weakened; the cooling trend of the ST in southwestern Tibet has turned into a warming trend; and the Qaidam Plateau has a significant warming trend. The surface of the Qinghai-Tibet Plateau has a warming trend, but the temperature change does not have obvious regional differentiation, similar to the other levels.

### Analysis of 20-cm ST characteristics of the Qinghai-Tibet Plateau

#### Regionalization of 20 cm ST

The ST at 20 cm was chosen as the research object because 1) 20 cm was basically the same as 5 cm, 10 cm, and 15 cm, and 20 cm was the boundary layer between the shallow soil surface and the shallow soil bottom layer^67^; 2) compared with 40 cm, the ST at 20 cm was greatly affected by climate factors such as temperature and precipitation but was less affected by geothermal conditions and had a more obvious response to climate change; and 3) the 20 cm ST directly affects the growth and distribution of soil organisms^68^. Therefore, 20 cm is very representative as the boundary between the shallow soil surface layer and the shallow soil bottom layer.

**Figure 7:**
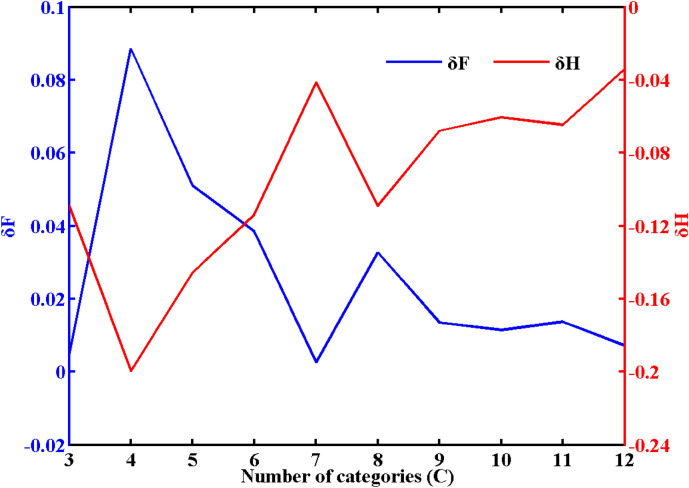
When m = 2.0, the entropy and distribution coefficient change with the number of categories ($C$).

**Figure 8:**
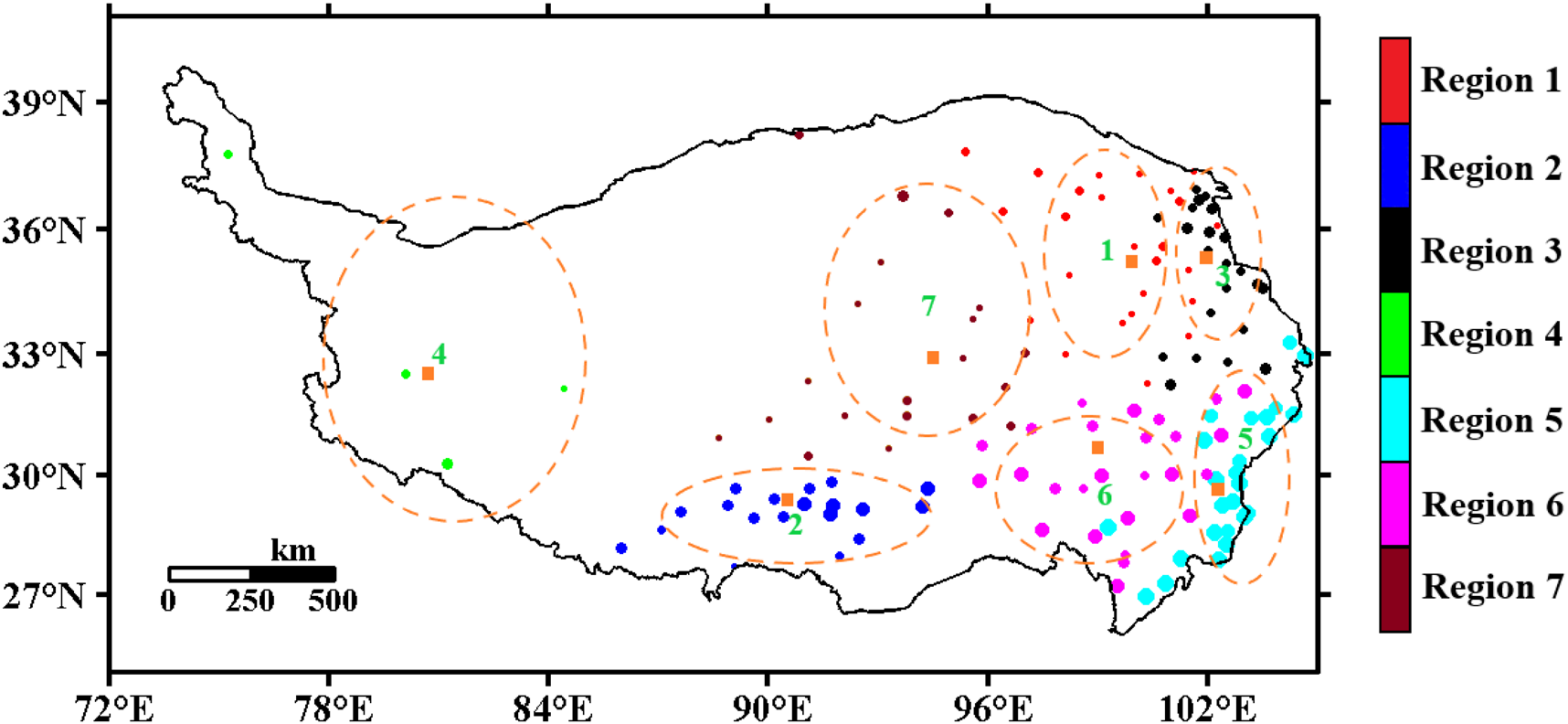
The 20 cm ST division of the Qinghai-Tibet Plateau (7 regions). The points are divided into four categories, from small to large: < 6 °C (cold region), 6–11 °C (subcold region), 11–16 °C (subwarm region), > 16 °C (warm region) (Software: MATLAB 2014, https://ww2.mathworks.cn/products/matlab.html).

Figure 7 shows the 20 cm ST division of the Qinghai-Tibet Plateau using the fuzzy C-means algorithm (FCM). In the process of FCM clustering, choosing the appropriate fuzzy index $m$ and the number of categories $C$ is the most critical. According to the research of Liu et al.^69^, the expected clustering effect can be achieved when $m = 2$. The choice of category number *C* is related to the change in entropy ($\delta H$, $H_{c} - H_{c + 1}$) and the change in the distribution coefficient ($\delta F$, $F_{c} - F_{c + 1}$). When the change in the distribution coefficient is the smallest and the change in entropy is the largest, *C* is determined. Using FCM, based on 20 cm ST at 141 sites on the Qinghai-Tibet Plateau, 7 regions were clustered. The classification results are shown in Fig. 8, and the environmental parameters of the cluster centers are shown in Table 2.

**Table 2:** Environmental parameters of the region center.

Region number	Region name	Longitude (°E)	Latitude (°N)	μ (°C)	σ (K)	Trend (K/10a)	Number of observation sites
Region 1	Northeastern of Plateau	99.9	35.4	5.2	0.60	0.46	25
Region 2	Southern Tibet	90.6	29.3	10.9	0.56	0.38	19
Region 3	Xining Plateau	101.9	34.9	8.9	0.48	0.33	22
Region 4	Qiangtang Plateau	81.1	32.6	7.6	0.61	0.61	4
Region 5	Western Sichuan Plateau	102.4	29.7	17.1	0.40	0.25	25
Region 6	Eastern Tibet Plateau	100.0	30.5	11.6	0.42	0.24	26
Region 7	Central of Plateau	93.8	32.4	6.0	0.49	0.35	20

The Qinghai-Tibet Plateau was divided into 7 regions according to the characteristics of the 20 cm ST, namely, the northeastern Tibetan Plateau, southern Tibet, the Xining Plateau, the Qiangtang Plateau, the western Sichuan Plateau, the eastern Tibetan Plateau and the vast Central Plateau. Lin et al.^70^ pointed out that the northeastern Loess Plateau is a sub-Arctic semihumid climate zone, with an average altitude of approximately 4000–4600 m, fewer than 50 days with a temperature ≥ 10 °C, and an average temperature of 8–10 °C in the warmest month. In Table 2, the average temperature of the 20 cm soil was the lowest, which was 5.2 °C; southern Tibet is a temperate and semiarid climate zone, and a large area is a wide river valley. The middle and upper reaches of the Yarlung Zangbo River are located in this region, with an average altitude of 3500–4500 m. The number of days when the temperature is ≥ 10 °C ranges from 50 to 150 days. The average temperature of the warmest month is 10–15 °C, while the average temperature of the 20 cm soil is relatively slightly higher, at 10.9 °C; the Xining Plateau is a temperate and semiarid climate zone, located in the eastern part of Qinghai Province, with complex terrain. The altitude of the valley area is below 2200 m, the average altitude is 2700–3300 m, the number of days with temperature ≥ 10 °C ranges from 50 to 150 days, and the average temperature of the warmest month is 11–13 °C. The average ST of 20 cm is 8.9 °C. The Qiangtang Plateau is a frigid semiarid climate zone with gentle terrain fluctuations; the average altitude is 4300–5100 m, there are fewer than 50 days with a temperature ≥ 10 °C, and it has an average temperature of 8–10 °C in the warmest month. The average ST of 20 cm is 7.6 °C; the western Sichuan Plateau is a temperate humid climate zone located on the eastern edge of the Qinghai-Tibet Plateau. The terrain fluctuates greatly, and the average altitude is 1500–3400 m. The number of days when the temperature is ≥ 10 °C ranges from 120 to 180 days. The average ST of 20 cm was the highest, which reached 17.1 °C. The eastern Tibetan Plateau is a temperate semihumid climate zone, with an altitude of approximately 2700–4000 m; additionally, there are approximately 50 days when the temperature is ≥ 10 °C, the average temperature of the warmest month is 13–16 °C, and the average 20 cm ST is 11.6 °C. The central Tibetan Plateau is a vast area, including most of Tibet and Xinjiang, with an average altitude of 4500–4800 m, fewer than 50 days with a temperature ≥ 10 °C, an average temperature of the warmest month of 6–10 °C, and an average of the 20 cm ST that is low, at 6 °C.

#### The characteristics of interannual variation at 20 cm ST in the different regions of the Qinghai-Tibet Plateau

**Figure 9:**
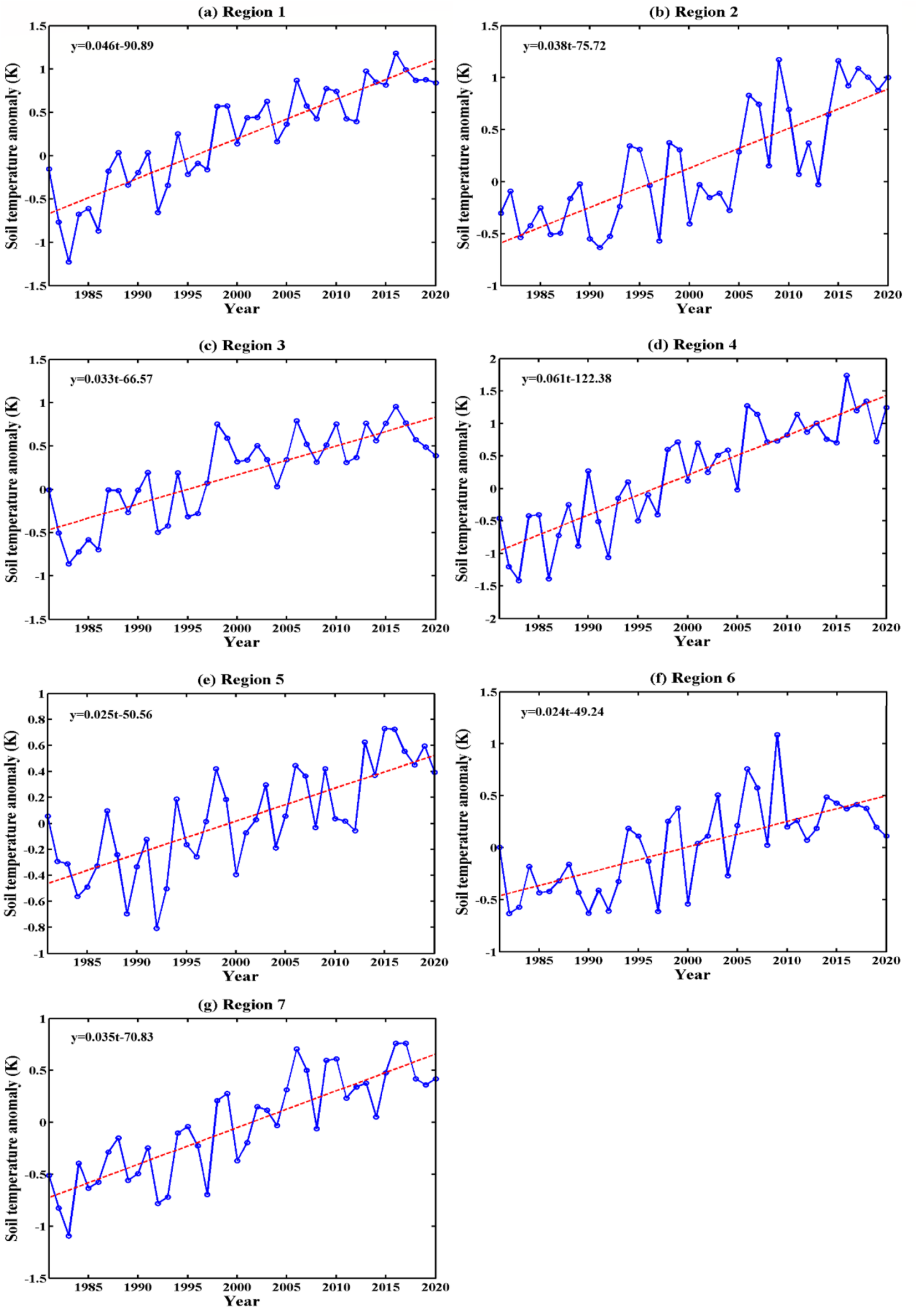
Interannual variation in the 20-cm ST anomalies in 7 regions of the Qinghai-Tibet Plateau (unit: K, ${\upalpha } = 0.01$).

Figure 9 shows the interannual variation in the 20 cm ST anomaly in 7 regions of the Qinghai-Tibet Plateau. According to Fig. 9 and Table 2, from 1981 to 2020, the ST at 20 cm in the 7 regions showed an obvious increasing trend, but the increase in each region was quite different. Linear fitting of the ST for each region showed that the interdecadal variability of region 1 was 0.46 K/10a, that of region 2 was 0.38 K/10a, that of region 3 was 0.33 K/10a, that of region 4 was 0.61 K/10a, that of region 5 was 0.25 K/10a, that of region 6 was 0.24 K/10a, and that of region 7 was 0.35 K/10a. The correlation coefficients with the months were 0.61, 0.51, 0.48, 0.64, 0.38, 0.38, and 0.59, all of which passed the confidence level *t* test with α = 0.001. The average values of different regions ranged from 5.2 (region 1, northeastern Plateau) to 18.1 °C (region 5, western Sichuan Plateau), and the average difference was nearly 12 K. The standard deviation of different regions ranged from 0.40 (region 5, western Sichuan Plateau) to 0.61 K (region 4, Qiangtang Plateau). Because the western Sichuan Plateau is located in a humid climate area, the shallow ST changed little, while the Qiangtang Plateau is located in an arid climate area, and the seasonal temperature difference is larger, so the shallow ST changed greatly, which was consistent with the obtained results. The results above indicate that the 20-cm ST increasing trend of the Qinghai-Tibet Plateau was obvious, and there were significant regional differences.

#### Spatial variation characteristics of the 20 cm ST on the Qinghai-Tibet Plateau

**Figure 10:**
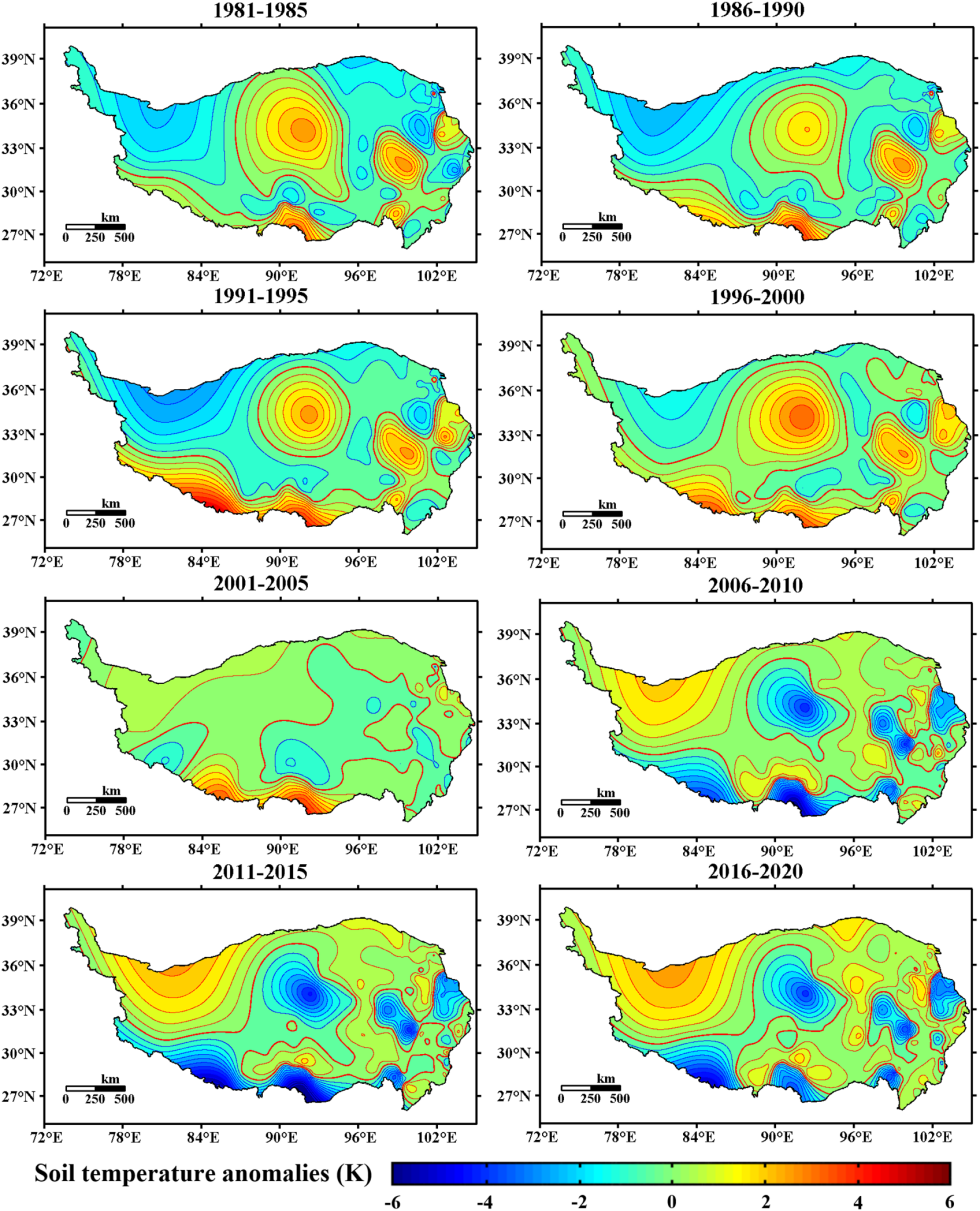
Spatial distribution of the 20 cm ST anomalies in the Qinghai-Tibet Plateau from 1981 to 2020 (average per five years, unit: K, Software: MATLAB 2014, https://ww2.mathworks.cn/products/matlab.html).

Figure 10 shows the spatial distribution of the 20 cm ST anomalies on the Qinghai-Tibet Plateau from 1981 to 2020. The figure shows that since 1981–2000, most areas of the Qinghai-Tibet Plateau have been in the cold stage, and the northwestern part of the Qinghai-Tibet Plateau is a strong and obvious center of negative ST in 20 cm, especially from 1991 to 1995. This negative anomaly center lasted for nearly 20 years.

In addition, from 1981 to 2000, there were four strong centers of positive anomalies on the Qinghai-Tibet Plateau, namely, the eastern part of the Qinghai-Tibet Plateau, the central-eastern part of the Qinghai-Tibet Plateau, the southern part of the Qinghai-Tibet Plateau, and the southwestern part of the Qinghai-Tibet Plateau. Among them, the positive anomaly of the southwestern part of the Qinghai-Tibet Plateau was strong in some years (such as 1981–1985 and 1986–1990), reaching above 2 K. In most years from 1981 to 2000, the positive anomaly of the 20 cm ST in the southern part of the Qinghai-Tibet Plateau was strong, with a value greater than 2.5 K (1991–1995). From 1981 to 2000, there was a positive anomaly center of approximately 1 K in central Tibet.

From 2001 to 2005, the positive anomaly center in southwestern Tibet disappeared, while the positive anomaly center in southern Tibet remained strong. In addition, the strong positive anomaly center in the central part of the Qinghai-Tibet Plateau disappeared in 2001–2005 after persisting for 20 years (1981–2000).

Based on the interannual variation in the 20 cm ST anomaly in Fig. 4(d1), the 20 cm ST anomaly across the Qinghai-Tibet Plateau changed from a negative value to a positive value since 2006. Figure 10 also shows that since 2006, the negative ST anomaly of 20 cm in most parts of the Qinghai-Tibet Plateau has begun to rise. Except for the scattered negative anomaly centers, soil temperature anomalies in most parts of the Qinghai-Tibet Plateau have changed between − 1 K and 1 K. It is worth noting that since 1981, the vast northwestern part of the Qinghai-Tibet Plateau is no longer a strong negative anomaly center that lasted for nearly 20 years.

The above analysis shows that the ST of 20 cm in the Qinghai-Tibet Plateau has had significant temporal and spatial changes. From 1981 to 2000, most areas of the Qinghai-Tibet Plateau were in the cold stage; in particular, the northwestern region had a strong negative soil temperature anomaly. Since 2006, the 20 cm ST anomaly has changed from a negative value to a positive value. From the perspective of spatial distribution, there was a strong negative ST anomaly center in the northwestern region from 1981 to 2000. Four positive anomalous centers generally exist in the Qinghai-Tibet Plateau, namely, the eastern part of the Qinghai-Tibet Plateau, the central-eastern part of the Qinghai-Tibet Plateau, and the southern part of the Qinghai-Tibet Plateau, the southwestern Qinghai-Tibet Plateau.

## Discussion and conclusion

### Discussion

Soil is an important component of the Earth's ecosystem^71^. Shallow soil refers to the soil layer within 50 cm underground. Shallow soil is also the main layer of life activities of animals, plants and microorganisms. The thermal properties of soil are key variables for the growth and decomposition of aboveground and belowground biomass^72–75^. Changes in soil thermal properties can alter soil enzyme activity, plant productivity, nitrogen uptake and the living conditions of soil microorganisms^76,77^. As one of the most important thermal properties of soil, soil temperature (ST) not only affects the chemical and biological processes of carbon and nitrogen in soil but also determines the quality of soil resources^78,79^. Shallow ST and its temporal and spatial changes directly or indirectly affect many processes occurring in soil, such as seed germination, root elongation, evaporation, storage and movement activities of microorganisms, nutrient cycling and many other dynamic soil processes^80–82^. Therefore, the analysis of shallow ST is very meaningful in the study of local ecological conditions and environmental characteristics. Currently, most studies on shallow ST on the Qinghai-Tibet Plateau are based on model results or reanalysis data from different sources. However, the Qinghai-Tibet Plateau has many deficiencies in the simulation and reanalysis results due to its complex geographical features and underlying surface conditions^46,47^. There are also many difficulties in the collection of observational data due to the harsh geographical environment.

**Figure 11:**
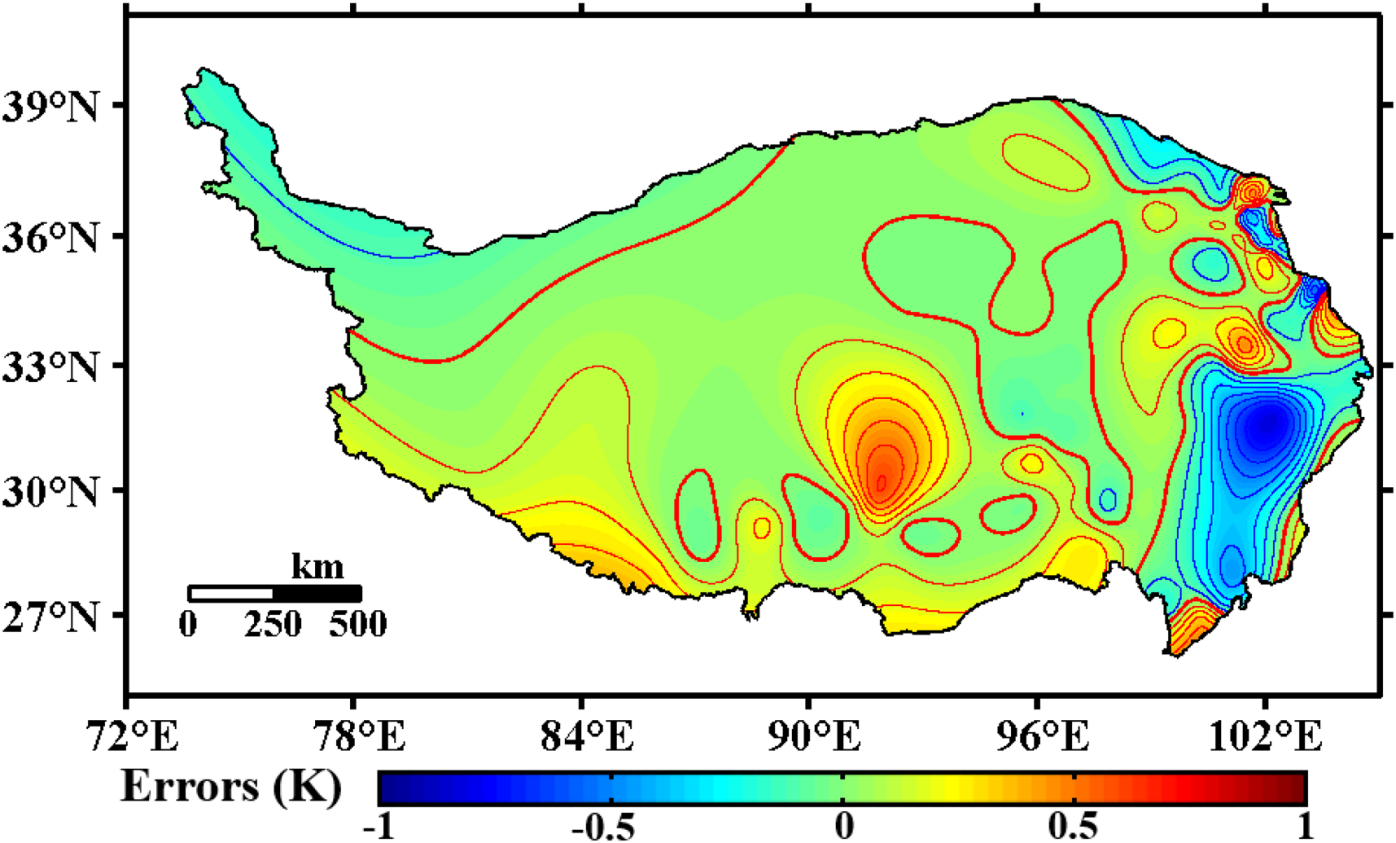
Errors of the “moving surface spline interpolation algorithm based on Green’s function” (annual average of 1981–2020, unit: K, Software: MATLAB 2014, https://ww2.mathworks.cn/products/matlab.html).

This work collected shallow ST observation data from 1981 to 2020 from most counties and districts on the Qinghai-Tibet Plateau. The data are relatively complete and abundant, and the time span is also long. The “moving surface spline interpolation algorithm based on Green’s function” was used to obtain grid data based on observational data with a spatial resolution of $0.25^\circ \times 0.25^\circ$. As shown in Fig. 11, the errors of the interpolation data in most regions were concentrated around $\pm 0.25$ K, the errors in the southeastern part of the Qinghai-Tibet Plateau reached − 0.5 K, and in some parts of the central plateau, the errors reached 0.3 K. In most regions, the errors of this interpolation method were less than 3%. It is worth noting that although the observation sites in the southeastern Qinghai-Tibet Plateau are denser, there are a large number of hot springs in the southeastern plateau, especially in the western Sichuan Plateau. Jiang et al.^83^ showed that the exothermic effect of hot springs has a very significant contribution to the air temperature and shallow soil temperature on the Qinghai-Tibet Plateau. The interpolation method we used has a good effect on the special topography of the Qinghai-Tibet Plateau, but it does not take into account the effects of other heat sources, such as the hot springs on the Qinghai-Tibet Plateau, resulting in a cold bias of 0.5 K within the southeastern Qinghai-Tibet Plateau. Eliminating the effect of other heat sources is also one of our next goals for improving this interpolation method.

**Figure 12:**
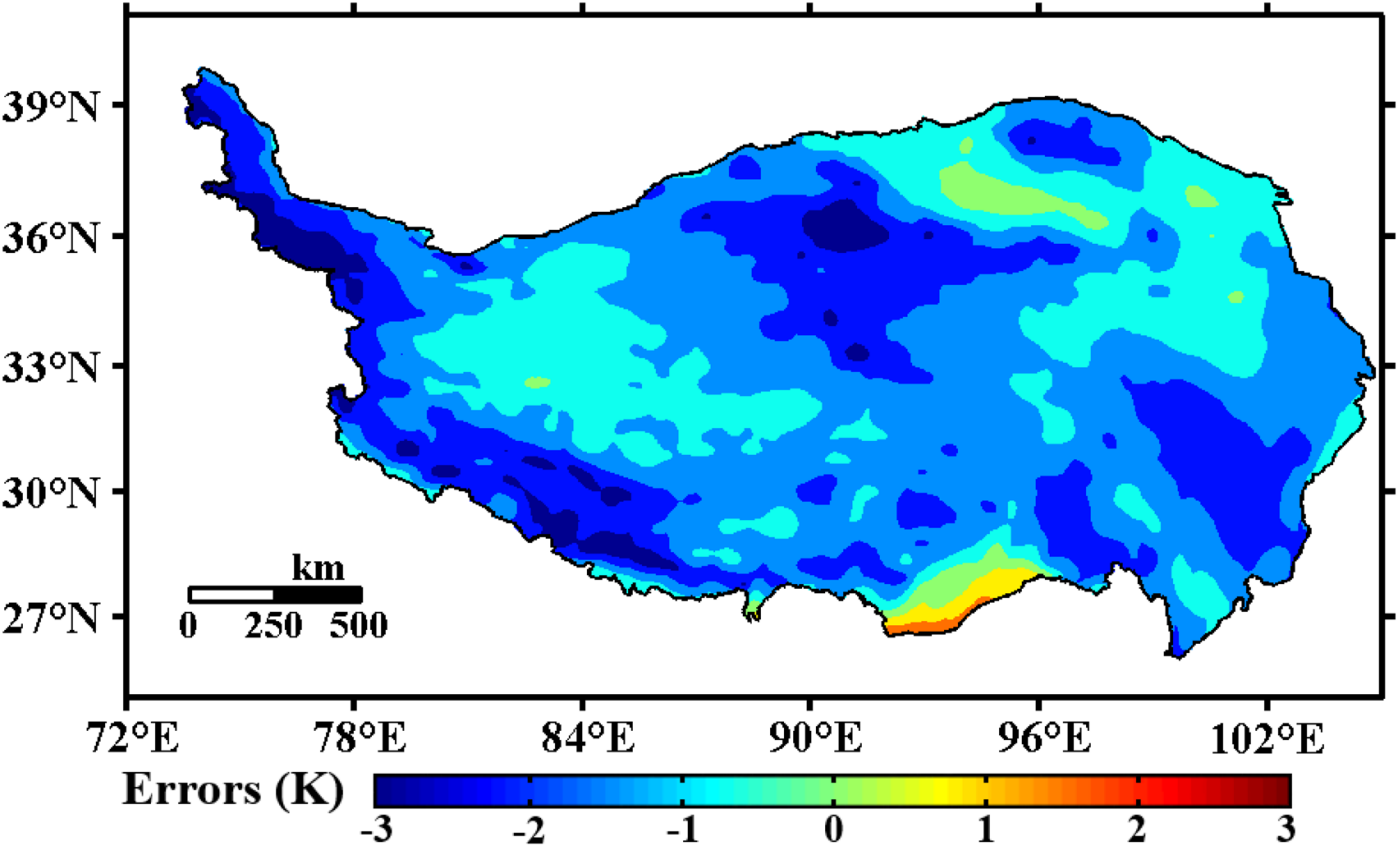
Errors of the ERA5 reanalysis data (annual average of 1981–2020, unit: K, Software: MATLAB 2014, https://ww2.mathworks.cn/products/matlab.html).

Figure 12 shows the error spatial distribution of the ERA5 reanalysis data. It can be seen from the figure that the reanalysis data are generally smaller than the observation data. In most parts of the central Qinghai-Tibet Plateau, the western part of the plateau, and the eastern part of the plateau, the errors reached − 1.5 K and even below − 2 K in some regions. Considering that grid data represented by reanalysis data are often used in many models, this work used ERA5 reanalysis data with a higher resolution and a longer time span and compared it with the interpolation results of soil temperature observations. The results showed that the cold bias in the central and western regions of the ERA5 reanalysis data was more significant. Chen et al.^84^ showed that the surface albedo-induced bias, surface cloud radiative forcing, clear-sky shortwave radiation, clear-sky downward longwave radiation, surface sensible heat flux, latent heat flux, and heat storage could cause the cold bias of the 2 m air temperatures as well as the surface temperature. This kind of cold bias was more significant in the western part of the plateau than in the east. This result was consistent with the shallow soil temperature results obtained from the ERA5 reanalysis data. It was also seen that the grid data obtained by using the ''moving surface spline interpolation algorithm based on Green’s function” were more credible when reflecting the shallow soil temperature of the Qinghai-Tibet Plateau and more accurate when describing the thermal properties and thermal conditions of the Qinghai-Tibet Plateau. The grid data obtained by using this method can help us understand the energy exchange in the earth-atmosphere system and improve land surface process models.

Additionally, some studies^85–89^ have shown that the Qinghai-Tibet Plateau is warming and wetting, with the annual average temperature rising significantly at a rate of 0.35–0.45 K/10a, and the closer the study period is, the more significant the warming trend is. In winter, the temperature increase is more obvious. The temperature increase in the northeastern part of the plateau is particularly significant, and the temperature increase rate can reach 0.49–0.56 K/10a. In our study, from 1981 to 2020, the soil temperature at 0–20 cm in the Qinghai-Tibet Plateau increased rapidly at a rate of 0.49–0.53 K/10a, and the soil temperature of the deeper 40 cm increased at a rate of 0.98 K/10a. The soil temperature increase in winter was also more obvious, indicating that the shallow soil temperature had a greater increase than the air temperature. In terms of the 20 cm soil temperature subregions, the 7 regions from north to south showed the characteristics of a gentle temperature increase in the south and a rapid temperature increase in the north, which was basically the same as the trend of air temperature increase.

Shallow soil is important in land‒atmosphere interactions. The mass and energy exchange between land and atmosphere is realized through the shallow soil. The understanding of the physical and chemical properties of shallow soil directly determines our understanding of land‒atmosphere interactions. Variations in shallow soil temperature are often influenced by changes in atmospheric conditions, and in turn, the continued development of shallow soil temperature anomalies can also affect the atmosphere. For example, Tang et al.^30^ pointed out that winter ST anomalies will affect precipitation in the following spring; moreover, Mahanama et al.^90^ pointed out that late spring ST has a good correlation with summer precipitation. To further reveal the influence of shallow soil temperature on air temperature and precipitation on the Qinghai-Tibet Plateau, the following work will further analyze the evolution as well as the abnormal signals of shallow soil temperature in the seven regions. This research helps reveal the mechanism of changes in the shallow soil temperature of the Qinghai-Tibet Plateau on air temperature and precipitation in China, with a view to contributing to the prediction of air temperature and precipitation in China.

### Conclusion

Based on observational data and ERA5 reanalysis data, this work studied the abnormal conditions of the shallow ST of the Qinghai-Tibet Plateau from 1981 to 2020 and further analyzed the temporal and spatial characteristics of the 20 cm ST to draw the following conclusions:

From the perspective of spatial distribution, the shallow ST of the Qinghai-Tibet Plateau had a trend of increasing from north to south, but Golmud and Qinghai had a higher ST area. In the vast northern and southern parts of the Qiangtang Plateau, the shallow ST was basically below 5 °C. From the perspective of temporal change, the temperature of the shallow soil on the Qinghai-Tibet Plateau had a clear increasing trend. The temperature increase of 0–20 cm (the surface layer of shallow soil) was roughly the same, which was quite different from that of 40 cm (the bottom layer of shallow soil).

The 20 cm ST on the Qinghai-Tibet Plateau gradually decreased from south to north and from east to west. The average ST in the western Sichuan Plateau, located in the southeastern Qinghai-Tibet Plateau, was the highest, with $T_{{20{\text{cm}}}} = 18.1\;^\circ {\text{C}}$. The temperature in the central and northern parts of the Qinghai-Tibet Plateau was the lowest, with $T_{{20{\text{cm}}}} = 4.9\;^\circ {\text{C}}$. Considering the 7 regions of the 20 cm ST, the warming trend was obvious, and there were certain regional differences. The average annual ST in different regions ranged from 5.2 (northeastern Plateau) to 17.1 °C (western Sichuan Plateau), with a difference of nearly 12 K; the standard deviation ranged from 0.40 (western Sichuan Plateau) to 0.61 K (Qiangtang Plateau), with a difference of 0.21 K.

Using the “moving surface spline interpolation algorithm based on Green’s function” to interpolate the observation site data, the errors of the obtained grid data were basically less than 3%, and these values were much smaller than the errors obtained from the ERA5 reanalysis data. This result shows that our interpolation data can better reflect the soil thermal conditions of the Qinghai-Tibet Plateau. This work is significant for understanding the characteristics of ST evolution and land‒atmosphere interactions on the Qinghai-Tibet Plateau and provides important data support for improving the underlying surface boundary conditions of models.

## Data Availability

The observational data used and analyzed during the current study available from the corresponding author on reasonable request. The reanalysis data generated and analyzed during the current study are available in the European Centre for Medium-Range Weather Forecasts (ECMWF) repository, (ERA5 hourly data on single levels from 1979 to present (copernicus.eu)).
